# Supplementary material for: Are carbon emissions trading and green financial instruments synergistic? -Comprehensive quantitative research based on content analysis
Source: PLoS One. 2024 Mar 7;19(3):e0298601. doi: 10.1371/journal.pone.0298601 (PMC10919601; doi:10.1371/journal.pone.0298601)
Supplement: S1 Data — (ZIP) [file pone.0298601.s001.zip › Data/地级市Z_F工作报告.zip!/╔┬╬≈/░▓┐╡╩╨/└┤╫╘╣1⁄2╓┌║┼í╛┬φ┐╦╩2╛▌═°í┐_2008.docx]

各位代表：

现在，我代表市人民政府向大会作工作报告，请予审议。并请政协委员和其他列席人员提出意见。

一、2007年政府工作的回顾

2007年，我们在中共安康市委的正确领导下，全面贯彻科学发展观，紧紧依靠全市人民，奋力实施突破发展，加快构建和谐安康，较好地完成了市二届人大二次会议确定的各项任务，在突破发展的征程上取得了新的重大进步。为我市近年来发展速度和效益最好的一年。

经济发展提速增效。完成生产总值189.9亿元，增长12.6%；全社会固定资产投资147.7亿元，其中地方固定资产投资99.1亿元，分别增长36.5%和39.3％；财政总收入20亿元，其中一般预算收入5.8亿元，分别增长56.4%和43.2%；社会消费品零售总额62.6亿元，增长17.7%；农民人均纯收入2256元，增长15.5%；城镇居民人均可支配收入8051元，增长17.4%；单位生产总值能耗预计下降4.5%。

“三农”工作得到强化。粮食总产104.8万吨，增长9.5%，为近十年最好水平。油料总产8.98万吨，增长15.2%。猪、羊、禽出栏量分别达到198.8万头、61.3万只、745.5万羽，分别增长9.6%、10.8%和9.1%。养蚕60.1万张，净增10万张，产茧2233.2万公斤，增长17.8%。茶叶总产532.2万公斤，增长13.3%。烤烟、魔芋、中药材等特色产业质量效益提升。一批龙头企业的成长拉动了产业基地建设，促进了重点农产品向特色工业品的转化。扶贫攻坚扎实推进，487个扶贫重点村出现明显变化，131个特困村当年农民纯收入高于全市增长水平，102个新农村建设试点村继续发挥示范带动作用。基础设施建设力度加大，灾后恢复重建成效明显。

重点项目扎实推进。绿色产业、基础设施、生态环境和社会事业25个重点项目年度投资基本完成，列入陕南突破发展的28个项目全部开工。汉江干流梯级电站建设全面展开，生物柴油一期工程动工兴建，三条百万吨水泥生产线开工建设。一批重点工业项目的竣工投产，支持了工业的快速增长，工业总产值跨上百亿新台阶。重点景区项目加快实施，生态旅游业出现强劲增长势头。交通建设全面推进，西汉高速宁陕段竣工通车，西康、陕川、十天高速公路建设进度加快；新建通乡油路446公里，铺筑通村水泥路3585公里，实现70%的乡镇通油路、55%的村通水泥路。安康至重庆、安康至武汉铁路复线加快建设，机场改扩建工程前期工作展开。330KV金州输变电等工程建成投运。丹江口库区上游水土保持项目正式启动，小流域治理、荒山造林、退耕还林、天然林保护等生态建设项目加快实施，汉江安康段水质保持国家Ⅱ类标准。

城镇建设力度加大。全市完成城建投资8.39亿元，比上年增长一倍。中心城市建设完成投资4.24亿元，增长39%。江北新区基础设施建设进度加快。汉江七里沟大桥合拢，安康大道路基全线贯通，滨江大道二期工程加快推进，花园大道、瀛湖干道正式开工。江南城区一批市政项目加快实施。香溪大道、桥西广场、高井路建设全面展开，雨污分流、强电入地项目有序推进。城区电子警察系统投入运行，“擦亮安康”活动取得初步成效，城市“脏乱差”的状况有所好转。县城建设步伐加快，集镇建设呈现新的亮点。

改革开放不断深化。全面完成国企改制任务，非公有制经济蓬勃发展。农村综合改革不断深化，乡镇机构改革全面完成。集体林权改革开始起步，政策性农业保险试点分步实施。国库集中支付、非经营性国有资产管理改革由市本级扩大到县区。实行了公务员工资制度改革，规范并兑现了公务员和事业单位工作人员的津补贴。招商引资取得新成果，全年共签订合同招商项目111个，引进资金72.15亿元，当年开工项目101个，到位资金14.21亿元。

社会建设全面进步。全市财政投入社会事业25亿元，增长39.2%。一批农业新技术和工业新工艺的应用，推动了经济发展方式的转变。新建和改扩建农村校舍23.5万平方米，排除危房校舍25万平方米，办学条件得到较大改善。公共卫生疾病防控和医疗救治体系不断完善，地方病、传染病防治成效明显，农村卫生院建设和村卫生室装备得到加强。人口和计划生育工作健康发展，全市保持低生育水平。广播电视通村入户工程启动，群众文化、文学创作、民间艺术繁荣活跃，全民健身迎奥运活动广泛开展。产品质量和食品安全专项整治行动取得成效。道路交通安全专项治理有力开展，全市安全生产形势明显好转。政法系统基层基础建设继续加强，社会治安综合治理和“平安安康”创建活动扎实开展，各类犯罪活动受到依法打击，社会大局稳定和谐。

改善民生取得实效。全市财政投入民生支出10.8亿元，增长40%。城镇新增就业12929人，基本消除了零就业家庭。城乡31.4万低收入群众全部纳入低保范围。新建农村敬老院109所、安置点65个，五保户集中供养率达到35%。农村义务教育“两免一补”政策得到全面落实，贫困家庭大学生全部资助入学。提高了离退休人员养老金和困难群众的低保标准。新型合作医疗提前一年覆盖全市农村，参合人数224.6万人，参合率91.5%。解决了2625户城镇和12904户农村困难群众的住房问题，因灾倒房的农户全部迁入新居。慈善协会、红十字会、残联的扶弱济困作用进一步发挥。

政府建设得到加强。坚持以科学发展观统领政府工作，突出经济发展，切实改善民生，强化公共服务，政府的公信力和执行力有了新的提高。进一步加强廉政建设，切实改进政风行风，认真执行人大及其常委会的各项决议，自觉接受人大、政协、舆论和公众监督。面对去年暴雨洪水和泥石流灾害，各级政府快速反应，靠前指挥，组织灾区群众全力抢险救灾，扎实开展灾后恢复重建，在战胜自然灾害中夺取了突破发展的新胜利。

各位代表！过去一年我们所取得的成绩，归功于科学发展观的正确指引，归功于全市人民的艰苦奋斗。在此，我代表市人民政府，向全市广大干部群众，向关心支持政府工作的各位人大代表、政协委员、各民主党派、离退休老同志和社会各界人士，向驻安人民解放军、武警官兵和公安干警，向所有关心、支持和融入安康建设的各界朋友，致以崇高的敬意和衷心的感谢！

在总结成绩的同时，我们必须清醒地认识到，我市的发展仍然是低水平的，经济结构没有大的变化，尤其是产业基础薄弱，工业经济落后，缺乏大产业、大项目、大企业的支撑；城镇化水平低，中心城市带动能力弱；环境保护任务艰巨，就业压力不断增大，财政收支矛盾突出，市场物价涨幅过快；城乡居民收入低、增长慢；一些干部思想解放不够，发展意识不浓，紧迫感不强；个别地方和部门形式主义、作风漂浮、落实不力的问题比较突出；政府工作人员的勤政廉政、服务水平与形势的发展和人民的期望还有不小差距。我们务必采取有力措施切实加以解决。

二、2008年工作的目标任务和总体要求

今年是全面贯彻党的十七大和十一届全国人大一次会议精神的第一年，也是我市在重要战略机遇期实施突破发展的关键一年。我们既面临着省上大力支持我市发展，加快破解交通等基础设施瓶颈制约的有利机遇，又面临着产业、工业、项目和工作不适应发展形势的严峻挑战，如果不迅速调整思路、改进工作、奋起追赶，势必坐失良机，掉队落伍。我们必须从滞后的现实中振奋起来，跳出安康看安康，着眼未来谋当前，以强烈的危机感、责任感和紧迫感，扎实做好今年的工作。

2008年政府工作的总体要求是：认真贯彻党的十七大精神，全面落实科学发展观，以解放思想为先导，以改革开放为动力，以突破发展为主题，以项目建设为支撑，以招商引资为抓手，扎实推进三大基础建设、六大支柱产业、八大民生工程，聚精会神抓发展，一心一意谋民生，尽职尽责保稳定，加快新型工业化、城镇化、农业现代化和人文化进程，努力促进经济社会又好又快发展。

经济社会发展的预期目标是：生产总值220亿元，增长12%；全社会固定资产投资180亿元，增长22%，其中地方固定资产投资120亿元，增长21%；财政总收入26亿元，增长30%，其中一般预算收入7.2亿元，增长24%；全社会消费品零售总额72亿元，增长15%；农民人均纯收入2480元，城镇居民人均可支配收入8860元，分别增长10%；居民消费价格总水平涨幅控制在4.8%左右；人口自然增长率控制在3.5‰以内；单位生产总值能耗降低4%；汉江安康段水质保持国家Ⅱ类标准。

实现今年目标，做好今后工作，必须牢固树立和自觉落实六大施政理念。

树立加快发展、突破发展的理念。必须真正把发展作为富民兴市的第一要务。牢牢扭住经济建设这个中心，不为困难所惧，不被干扰所惑，更加坚定地实施突破发展、加快发展。

树立产业兴市、项目第一的理念。必须集中力量破解项目建设难题，强力推进产业建设和工业发展，把抓项目作为检验工作成效、衡量领导水平的首要标准。

树立改革开放、开拓创新的理念。必须进一步解放思想，冲破一切制约发展的思想障碍和体制束缚，把改革创新精神贯彻到加快发展的各个环节。

树立以人为本、民生至上的理念。必须全心全意为人民服务，更加注重改善民生，更加注重公平正义，更加注重人的全面发展，把政府工作根植于人民群众的沃土之中。

树立生态立市、永续发展的理念。必须坚持全面协调可持续发展，保护建设好安身立命的生态环境，切实转变经济发展方式，走生态文明发展之路。

树立艰苦创业、埋头狠干的理念。必须用“三苦”精神改造我们的作风，坚持苦字当头，狠字当先，勤奋为首，把加快发展的坚定信念落实在苦干实干狠干的具体行动上。

三、奋力夺取突破发展的新胜利

跟进全省加快发展的新形势，顺应全市人民盼望发展的新期待，把握经济发展的新趋势，统筹工业与农业、产业与基础、城市与农村、当前与长远、市内与市外，努力把经济发展搞上去。

(一)突出县域特色经济，扎实推进新农村建设。以“一村一品”为抓手，加快培育富民强县的主导产业。按照“稳粮、强牧、壮特、增收”的思路，坚持强村大户、龙头牵引、集约经营，在稳定粮食总产、确保粮食安全的基础上，集中主攻畜牧、蚕桑、茶叶、魔芋、烤烟、蔬菜、药材和木本油料等产业的规模经营，建立稳固的工业原料基地和农民持续增收的可靠基础。稳定劳务输出，提高输出质量。继续增加财政对农村建设的投入，加强农田水利建设，抓紧病险库塘除险加固，加大土地开发复垦力度，加快农村道路、供电、沼气、人居环境等基础设施建设，深入推进农业综合开发和扶贫攻坚。以“一县一业”为主导，推动县域经济突破发展。深化对县情、区情的认识，找准发展优势，明确主攻方向，大幅提高主导产业的规模效益，大幅提高工业在经济总量中的比重，大幅提高县域经济的核心竞争力。充分发挥月河川道的区位优势，加速形成要素集中、产业聚集、效益最佳、充满活力的产业经济带。大力支持基础条件较好的旬阳、汉阴、石泉、平利四县做强产业、做大工业，实现率先突破发展。旬阳要全力向全省经济十强县冲刺，汉石平三县要矢志进入全省县域经济中上水平。全力帮助汉滨区加速产业聚集，通过谋划和新上一批大企业、大项目，提升经济总量，壮大工业实力，增加财政收入，真正成为产业大区、经济强区。积极鼓励紫阳、白河、岚皋、宁陕、镇坪五县提速增效，敢于向条件较好的县区叫板，做大做强支撑县域经济发展的特色产业，使发展速度、人均水平在全市领先。

(二)大力推进产业聚集，加快新型工业化进程。进一步统一思想认识，从谋划项目做起，从破解难题入手，做大做强绿色能源、安康丝绸、富硒食品、金州矿产、秦巴医药五大工业支柱，强力推进新型工业化进程。确保三个百万吨大型水泥、生物柴油一期、500吨捻丝、双稀沃氏、万吨调味品、2万吨氯化钡、5万吨富硒矿泉水、泸康酒技改等10个投资3000万以上的项目竣工投产；加快蜀河电站、重型汽车制动器总成、湘贵锰业二期、旬阳50万吨铅锌选矿、平利30万吨铅锌选矿、万吨纳米氧化锌、五氧化二钒扩建、烟厂技改、镇坪和平利两条腊肉精加工生产线等10个投资3000万以上项目的建设进度；抓紧旬阳电站、白河电站、南江河流域梯级开发、燃料乙醇、20万吨饲料、旬阳和汉阴两个3000吨魔芋精粉、木瓜浓缩汁、优质板材硐采、桑椹醋饮等10个投资2000万以上的项目开工建设；推进两个大型火电、10万吨锌冶炼、10万吨锌焙砂二期、旬阳铁矿采选、5000吨工业硅、昊华钡盐二期、180万米真丝绸、钒矿采选、铜粉采选等10个投资亿元以上项目前期工作。全年实现工业产值140亿、工业增加值50亿以上，力争增速超过20%。要更加突出工业项目建设，从市县区政府班子成员和经济综合部门领导做起，人人承担招商引资任务，个个领衔工业项目建设，真正形成上大项目、办大工业、抓大品牌的浓厚氛围。实行项目建设、融资支持、工业用地的从优政策，培育一批产值超1亿、过5亿、上10亿的工业企业，带动中小企业的快速成长。高度重视产业聚集平台建设，进一步激活工业园区。紧紧抓住省上支持县级产业园区建设的机遇，力争建设10个各具特色的县级工业小区。市政府将进一步完善工业项目推进机制，建立工业发展联席会议制度，积极解决项目建设中的具体问题，扎实推进工业的快速发展。

(三)发展壮大中心城市，努力提升城镇化水平。坚持以规划为统领，准确把握城市建设的方向和品位；以特色为基调，彰显山水园林城市风格和建筑文化魅力；以“双创”为抓手，全面提升城市建设和管理水平。今年要聘请国内知名的城市规划专家，组织本市对历史文化研究具有一定造诣的各界人士，联合进行安康中心城市总体规划修编。加强对县城和村镇规划的指导，要高水平规划，高品位建设，形成各自的独特风格，不盲目建设，不急于求成，不留下遗憾。中心城市、县城和重点集镇都应按照风景名胜区和旅游目的地的要求进行规划和建设，努力形成安康独具特色的山水园林城镇体系。认真组织实施中心城市55个建设项目，完成年度投资6.5亿元。江北新区建设的重点是建成安康大道、汉江七里沟大桥、滨江大道二期、花园大道等工程，加快瀛湖干道建设进度，开工建设长岭路、黄沟路、寇家沟亲水广场等项目，完成汉滨区级机关和大学园区北迁规划编制和征地工作。江南城区建设重点是建成香溪大道、桥西广场、高井路一期等工程；开工建设西坝防洪、南环干道、南环东路、新城片区改造、市民广场等项目。扎实做好城东汉江大桥、滨江大道三期、巴山路改造、长岭北路、天然气供输管网等项目前期工作。着眼于城市的未来发展，着力提高新建项目的品位。每项重点工程的建设方案和命名都要充分征求广大市民的意见和建议。要把产业聚集和工业发展作为城市建设的重点，大力发展特色产业、新型工业、现代服务业，把中心城市建设成为带动月河川道率先发展的核心板块，形成融入关中经济区和连接多数县城的“一小时经济圈”。今年在深化“擦亮安康”活动的基础上，启动安康中心城市创建省级卫生城市和国家优秀旅游城市工作，鼓励各县争创省级卫生县城，汉阴、宁陕、平利三县要率先达标。组织开展卫生乡镇和村庄创建活动，以“双创”推进城镇建设管理水平的全面提升。

(四)打造生态旅游品牌，促进特色服务业发展。紧紧抓住西汉高速公路通车、西康高速公路即将建成和省上大力扶持的机遇，以秦巴风情、汉水神韵、金州美食、绿色安康为主题，抓紧旅游总体规划评审，突出岚皋、汉滨、宁陕三个旅游强县区建设，打造以南宫山为核心，连接中心城区、瀛湖的主打品牌景区，培育以秦岭南麓自然生态为重点融入秦岭国家植物园的森林体验一日游，以瀛湖为重点连接汉江库区水面的汉水风光两日游，以南宫山为重点融山亲水连城的巴山风情三日游三条精品线路，大力推进一批具有鲜明特色的重点景区建设。舍得拿出最好的资源、最佳的景区、最优的政策，吸引大企业开发、建设和管理。加快宣传促销力度，提升旅游业发展的整体水平。力争五年内实现年接待游客1000万人次的目标。进一步办好“龙舟节”等节庆活动。推进交通运输、物流配送、经贸会展、宾馆餐饮、文化娱乐、银行保险、信用担保、中介咨询、现代信息等服务业的发展。

(五)全力推动交通建设，加快破解瓶颈制约。创造各种有利条件，确保西康高速年内贯通，明年上半年全线通车；加快安康至陕川界高速公路建设，全面开工十天高速安康段工程。继续加大地方公路建设力度，全面完成国省道大中修任务，加快西汉高速两河连接线、小康高速东镇连接线建设，力争安岚二级公路年内开工。进一步加快县乡村道路建设，实现80%的乡镇通油路，80%的村通水泥路。大力支持武汉至安康、安康至重庆铁路复线建设，力促西康铁路二线提前开工。抓紧安康机场改扩建项目的核准和筹资工作，务求今年正式开工。

(六)坚持生态立市战略，转变经济发展方式。增强全社会生态文明意识，保护好我们赖以生存和发展的秀美山川，担负起一江清水供北京的政治责任和道德义务。继续抓好退耕还林、荒山造林、天然林保护、小流域治理、生态移民工程，扎实实施丹江库区及上游水土保持项目，加快安康城区雨污分流管网、江南污水处理厂二期工程建设，开工建设江北和白河污水处理厂。抓好“引汉济渭”工程的援建工作。坚持结构调整、工程治理、环境监管相结合，提高“三同时”执行率，杜绝新的污染源。以节能、节水、节地和资源综合利用为重点，大力发展循环经济，坚持把节能降耗减排的各项控制性指标落实到县区、行业和企业。加强土地、河流、矿产、森林等资源的保护和节约利用。以先进技术改造提升传统产业，大力推行墙体材料革新和建筑节能。深入开展节能减排全民行动，完善节能减排的奖惩机制，确保实现全年节能减排目标。

四、扎实推进以民生八大工程为重点的社会建设

社会建设与人民的幸福安康息息相关。我们必须在加快经济发展的基础上，从解决人民群众最关心、最直接、最现实的利益问题入手，保障和改善民生，推动和谐安康建设。

实施民生八大工程。整体推进教育、就业和再就业、公共卫生、社会保障、住房保障、农村基础设施、公益性文化体育设施、城乡社区服务体系建设八大民生工程，是省委、省政府的重大战略部署，是全面加强社会建设的民心工程。市政府把民生八大工程的实施作为造福全市人民的大事来抓，及时与省上进行了投资与项目的衔接，制定了4055个项目、总投资114亿元的五年规划。在总投资规划中，中省投资58亿元，市县区财政配套20亿元，社会投资36亿元。采取投资与项目配套、计划与县区对接的办法，确定了今年21.1亿元的项目实施计划,其中，中省投资14.98亿元,市县区财政投入2.77亿元，社会投入3.35亿元。具体计划已印发给各位代表。目前，资金保障、项目安排、推进机制已经落实,实施工作正在有效展开。在确保整体推进的基础上，市政府从中直接抓好十件实事：①对全市未升学的初高中毕业生实行全部免费职业技能培训；②新建和改扩建农村义务教育、高中教育、特殊教育教学生活用房12万平方米；③解决1.3万新增劳动力和下岗职工的就业和再就业问题;④将新型农村合作医疗的政府补助标准由人均40元提高到80元；⑤建立全市城镇居民基本医疗保险制度，实行城镇非从业人员、中小学生、少年儿童基本医疗保险的全覆盖，政府对参保人员每人每年补助80元；⑥新建3所县区中心敬老院、18所乡镇敬老院、改扩建30所乡镇敬老院，使农村五保户集中供养率达到50％；⑦解决6000户城镇特困户的住房和农村30400名贫困人口的安居问题；⑧力争新修通村水泥路3000公里，解决40万农民的出行难问题；⑨开工建设西坝防洪保安工程，根治危及西坝片区的水患之害；⑩解决16万农民的饮水安全问题，减少10万贫困人口。我们有信心、有能力如期完成民生八大工程的建设任务，显著改变社会建设滞后的现状，努力把人民群众祖祖辈辈渴望的养老就业有保障、家家户户有住房、就医上学有保证、饮水清洁保健康、道路便捷又通畅的夙愿变为美好的现实。

全面发展社会事业。坚持教育优先发展，积极开展以教育强县为重点的创建工作，旬阳、白河、石泉要率先达标。继续开展教育强乡镇创建活动。推动幼儿教育、义务教育、高中教育、职业教育、高等教育、成人教育、特殊教育协调发展，办人民满意的教育。高度重视和关心留守儿童教育。在打牢义务教育基础的前提下，重点加强职业教育和高中教育，提高劳动者素质，使更多的安康子弟通过大学教育走出大山，到安康之外去创业发展，共享全国乃至世界发展的成果。建立自主创新的激励机制，加快科技成果和先进技术的引进、转化和推广力度。高度重视项目领军人物、工业专门人才、科技研发人员和企业家队伍的培养。进一步加强公共卫生、农村卫生和医疗服务体系建设，继续加强儿童计划免疫和重大传染病、地方病防治，推行农村妇女免费住院分娩，落实贫困母亲住院分娩补助政策。坚持以稳定低生育水平为目标，促进人口与计划生育工作健康发展。继续完善城镇职工基本养老保险和基本医疗、失业、工伤、生育保险制度，逐步提高城乡居民最低生活保障标准和困难群体医疗救助标准。积极发展慈善、老龄和残疾人事业。大力发展文化、新闻、广播、影视、传媒、体育事业，深入开展群众性精神文明创建活动，促进文化事业的大发展大繁荣。

切实加强社会管理。扎实开展解决信访积案的集中行动，进一步加强信访工作。强化社会治安综合治理，依法打击各类违法犯罪，强化公安基层基础工作，深入开展“平安安康”创建活动。毫不放松以道路交通为重点的安全生产，坚决遏制重特大事故发生，促进安全生产形势持续好转。有效保障食品药品安全，切实抓好“菜篮子”工程，建立健全粮油肉等生活必须品储备和流通体系，确保市场繁荣活跃，控制物价过快上涨。大力支持少数民族聚集区的经济社会发展，依法加强宗教事务的管理，重视以民兵整组为重点的国防后备力量建设，深入开展“双拥”活动。切实加强对台工作。突出抓好汉江防汛和地质灾害防御。提高应对自然灾害、事故灾难、公共卫生和社会安全事件的能力。加强政府自身建设

新的形势和新的任务，对政府自身建设提出了新的更高要求。我们要在科学发展观的指引下，深入解放思想，创新政府工作，提高行政能力，努力建设顺应时代要求、适应科学发展、符合人民意愿的政府。

深入解放思想。扎实开展以突破发展为主题的解放思想大讨论活动。从市政府领导成员做起，从各部门一把手抓起，带动全市广大干部的思想大解放。着力转变不适应加快发展的思想观念，着力解决制约加快发展的突出问题，着力增强加快发展的危机感和紧迫感。在解放思想中统一思想、明确重点、实施突破。要走出去开阔视野，虚心学习外市加快发展的先进经验，在比较中寻找差距，制定出奋起改变落后的具体措施。要沉下去调查研究，紧紧围绕事关全局发展的重大问题，积极探索加快发展的新思路、新途径和新举措。

大胆改革创新。认真落实支持非公有制经济发展的政策措施，鼓励全民创业。巩固农村综合改革成果，健全土地承包经营权流转机制，鼓励发展多种形式的适度规模经营。采取有效措施积极化解乡村债务。稳步推进人工商品林采伐和集体林权制度改革。推进新型农村金融机构发展。积极开展资源有偿使用制度和生态补偿机制的调查研究，更多地争取国家支持。深化开发性金融合作。认真研究资本市场，促进有条件的企业上市。完善国库集中支付、非经营性国有资产管理、政府采购、财信担保等制度。强化税收征管，严格预算约束，壮大地方财力。按照国务院和省政府的部署，逐步健全职能有机统一的大部门体制，推进政府职能转变。把招商引资作为扩大开放、推动发展的第一要务，实行领导招商、干部招商、全民招商、以企招商，建立健全招商引资目标责任制和推进机制。拿出好资源、制定好政策、创造好环境，重点招引省内和长三角、珠三角等发达地区的大企业来安康投资建设。认真研究产业政策和市场信息，主动承接东部产业向西部转移、沿海经济向内陆转移的机遇，大力引进劳动密集型、技术密集型项目和企业落户安康。

狠抓工作落实。科学发展观创新了政府的施政理念和工作方式，要求我们更好地履行经济调节、市场监管、公共服务和社会管理职能，加快建设服务型政府。必须坚决防止脱离群众的官僚主义、主观主义、形式主义，崇尚实干、力戒空谈、讲求效率；必须坚定地把加快发展放在政府工作的首位，确定的目标毫不动摇，认准的事项盯住不放，决定的工作立说立行；必须实行严格的目标责任考核，从领导机关到基层单位，从领导干部到工作人员，从各级政府到工作部门，都必须把今年的目标任务、经济发展、民生工程落到实处。

坚持清正廉洁。始终保持不骄不躁、艰苦奋斗的作风，带头执行廉洁自律的各项规定，自觉履行廉政建设责任制，扎实推进惩治和预防腐败体系建设。推进法制政府建设，依法规范政府行为，依法管理社会事务，依法加强公务员队伍建设。厉行勤俭节约，狠刹奢侈浪费，切实改进政风行风。自觉接受人大的依法监督、政协的民主监督、媒体的舆论监督和人民的公众监督，善于吸收人民群众的批评、意见和建议，更好地改进我们的工作。在这里，我代表市政府全体组成人员，向全市人民庄严承诺：切实做到严于律已、廉洁奉公、干干净净，倾心尽力为安康的发展、为300万人民的福祉恪尽职守、勤奋工作、不辱使命。

各位代表！抚今追昔，我们深感肩负的使命神圣而光荣；展望未来，我们对安康的光明前景充满信心。让我们高举中国特色社会主义伟大旗帜，在科学发展观的指引下，认真落实市委的决策，紧紧依靠全市人民，只争朝夕，奋力拼搏，调动一切积极因素，冲破一切艰难险阻，谱写安康又好又快发展的新篇章！
